# Trainee as trainer in paediatric laparoscopic appendicectomy

**DOI:** 10.1308/rcsann.2024.0032

**Published:** 2024-05-24

**Authors:** L Henderson, N Patwardhan, H Dagash

**Affiliations:** Leicester Royal Infirmary, UK

**Keywords:** Laparoscopic surgery, Teaching, Training, Appendicectomy

## Abstract

**Background:**

Intraoperative teaching is an essential surgical skill, but there is little literature regarding trainees acting as trainers; we characterised these cases in paediatric laparoscopic appendicectomy.

**Methods:**

This is a retrospective review of casenotes over two years (2015–2017) in a single tertiary paediatric surgical centre in the UK. Operating were: paediatric surgery Core Trainees (CT) (postgraduate year (PGY)3–4), Junior Registrars (JR) (PGY5–6) and Senior Registrars (SR) (PGY7+); collectively described as trainees.

**Results:**

A total of 53 (20.7%) of 256 appendicectomies were trainee as trainer (TT) cases; 22 cases (41.5%) were performed by a CT supervised by a Registrar, and 31 (58.4%) by a JR supervised by a SR. Among the cases, 17 (32.1%) were complex, 47 (88.7%) were in working hours (8am–5pm), and 50 (94.3%) took place Monday to Friday. Median (interquartile range) duration of surgery was 65 (52–77) minutes. In the first year, 60 (47%) appendicectomies were performed by JRs. JR 1 was TT in three cases (8.8%) and JR 2 in five cases (19.2%); in all cases, the learner was a CT. Overall, there were 26 (10.6%) negative appendicectomies, 8 (3%) conversions, 19 (7%) readmissions within 30 days of discharge and 3 (1.1%) required reoperation; there was no statistically significant difference in complications between TT and non-TT cases.

**Conclusion:**

Laparoscopic appendicectomy is an excellent model for trainees to act as trainer; case selection included simple cases during daylight hours. Our outcomes are comparable with published literature, suggesting that this teaching method is safe for patients.

## Introduction

Teaching operative skills is an essential part of a surgeon's role. Providing safe clinical supervision, and effective education and training form part of the Generic Professional Capabilities required by all doctors, as described by the General Medical Council in the UK.^1^

As the number of hours in surgical training reduces in most countries, newer ways of working become necessary to allow competency to be achieved.^2,3^ Appendicectomy represents the commonest emergency procedure performed in children, with around 12,000 cases per year in the UK.^4^ Training another trainee is recognised as the highest level of entrustable professional activity, as reflected in trainee logbooks.^5^

UK Core Trainees (CTs) in Surgery are in their third and fourth postgraduate years (PGY) (PGY3–4), after completing two years as foundation doctors (PGY1–2). Registrars have progressed to subspecialise in paediatric surgery, for example, and complete a minimum of six years (PGY5–10) before applying for a Consultant post. In this article, we refer to Junior Registrars (JRs) in their first two years of Registrar training (PGY5-6), and Senior Registrars (SRs) with more than two years' experience (PGY7+). All of the above are collectively referred to as Trainees. Consultants have completed training.

We chose laparoscopic appendicectomy as a common example of intraoperative teaching, to characterise trainee as trainer (TT) cases.

## Methods

Our centre provides tertiary paediatric surgical care with involvement of trainees in all parts of the patient management pathway. The decision to operate is taken by the on-call Registrar, with oversight from the Consultant. Registrars undertake a weekly laparoscopic training programme, delivered by one Consultant with a special interest in minimally invasive surgery. This involves set tasks performed on a box trainer under 2:1 supervision. JRs are given additional exposure to appendicectomies during their early learning, with supervision from SRs or Consultants. It is not mandatory for a Consultant to be present during appendicectomy in our centre.

A prospective audit was performed of all appendicectomies and diagnostic laparoscopies for abdominal pain performed in children aged <16 years, with data presented on a monthly basis. This dataset was used to analyse the operator and assistant level, identifying TT cases. TT cases were analysed for who teaches, who is taught, time of day, day of week, complexity of cases, and operative time. JR experience was also analysed separately. Histology, length of stay, readmission within 30 days of discharge and complications were also analysed. Fisher's exact test was used to compare groups, with a *p*-value of <0.05 used to signify statistical significance.

## Results

### Overview

Over a two-year period (1 April 2015–31 March 2017), 256 appendicectomies were performed, 11 during diagnostic laparoscopies. All cases were commenced laparoscopically, with eight conversions to open (3%). The median age at operation was 11 (range 1–16) years. A total of 13 patients were managed nonoperatively and were excluded; 53 patients (20.7%) underwent abdominal ultrasound scan and no patients received abdominal computed tomography scan.

Median length of stay was 93.5 (range 8–458) hours.

### Who operated? Who was teaching?

Overall, 201 (78.5%) appendicectomies were performed by a Registrar, 33 (12.8%) by a Consultant, and 22 (8.6%) by a CT.

Appendicectomies performed by a trainee with no Consultant present contributed 185 (72.3%) of the operative cases. Of these, 140 (75.6%) were performed by a Registrar assisted by a CT, and 45 (24.3%) cases were TT.

Overall, 53 cases (20.7%) were TT; 22 cases (41.5%) were performed by a CT supervised by a Registrar, and 31 cases (58.4%) were performed by a JR supervised by another Registrar. In eight (15.1%) of these cases, a Consultant was unscrubbed in theatre to observe.

In the first year, where two new JRs started in the department, JR 1 performed 34 appendicectomies, and JR 2 performed 26, totalling 47% of all appendicectomies performed in that year. Towards the end of that year, JR 1 was TT in 3 cases (8.8%) and JR 2 was TT in 5 cases (19.2%); in all cases the learner was a CT.

### What was the case complexity?

Overall there were 245 appendicectomies: 114 (46.5%) with simple appendicitis, and 102 (41.6%) with complex (gangrenous or perforated) histology. There were 26 negative appendicectomies (10.6%). Two patients had acute pinworm infestation, and one incidental finding of neuroendocrine tumour of the appendix. Of the 11 patients who underwent diagnostic laparoscopy, nine had a normal appendix, one acute pinworm infestation, and one had acute appendicitis.

Of the 53 TT cases, 8 (15.1%) were normal appendices, 28 (52.8%) were simple and 17 (32.1%) were complex appendicitis (either gangrenous or perforated), based on histology reports. The rate of complex appendicitis was significantly lower in the TT group (*p*=0.020).

There were seven (3.4%) conversions to open in the non-TT group and one (1.9%) in the TT group (*p*≥0.999).

In the non-TT group, there were 34 patients (16.9%) under five years of age, compared with three patients (5.6%) in the TT group (*p*=0.047).

### When did the cases take place?

Of the non-TT cases, 121 (60.2%) took place in normal working hours, and 80 (39.8%) took place out of hours. A total of 135 cases (67%) took place Monday to Friday, and 66 (32.8%) at the weekend. Of the 53 TT cases, 47 (88.7%) took place in normal working hours (8am–5pm), and 6 (11.3%) were out of hours (*p*<0.001). A total of 50 (94.3%) cases were performed Monday to Friday, and 3 (5.7%) at the weekend (*p*<0.001).

### What was the operative time?

Overall median (interquartile range) duration of surgery was: 61.5 (46–80) minutes for a Consultant, 64 (52–81) minutes for a Registrar, and 67 (55–76) minutes for a CT.

Overall duration of surgery for TT cases was 65 (52–77) minutes. This was 72 (62–77) minutes for a CT assisted by a Registrar, and 62 (51–84) minutes for a JR assisted by an SR.

### Complications

In the non-TT group, 13 patients (6.4%) were readmitted within 30 days of discharge, and three patients (1.1%) required reoperation. In the TT group there were 6 (11.3%) readmissions (*p*=0.240) and no reoperations (*p*≥0.999).

## Discussion

Our results reveal that in one in five of all appendicectomies performed at our institution, a trainee is acting as a trainer. In a US study, 25% of a Resident's (equivalent of a Registrar's) time has been estimated to be spent teaching or supervising medical students or other trainees, although the proportion of time spent on intraoperative teaching has not been studied.^6^ A UK study of 311 General Surgical trainee electronic logbooks showed that JRs perform a mean of 26 appendicectomies in their first year.^7^ This is very similar to the findings for our two JRs (JR 1=34, JR 2=26). In our department of seven Registrars, an expected mean number of cases would be 18 per year, suggesting that the junior members are given preference to undertake appendicectomies by their colleagues. The authors also showed that TT experience in laparoscopic appendicectomy increased with level of training, from 12% in the first year, to 30% by the sixth year.^7^ Our JRs were able to achieve 9% and 19% TT cases in their first year.

Near-peer teaching can enhance trainees' self-efficacy and has been shown to improve overall job satisfaction.^8^ It is based upon social constructivism, where knowledge is built through social interactions in the learning environment.^9^ A Likert scale for evaluation of intraoperative teaching was tested in trainees taught by Consultants, and found that trainers with fewer than five years’ experience as a Consultant received the highest ratings. The authors suggest this may be due to a more similar experience in style of training, or trainees feeling more comfortable learning from someone they perceive to be ‘similar’ to themselves.^10^ Another study identified attributes of a successful surgical trainer, including willingness to let a junior operate, and balancing supervision with independence.^11^ Potential barriers to near-peer teaching are the perception that teaching can be burdensome, or insecurity in teaching someone with a similar experience level.^8^ A Swiss study asked European Registrars to rate the influence of a variety of teaching resources on their learning; they found that whereas other Registrars were rated first on the ward, they were rated fourth in the operating room, suggesting that TT is still an underused resource.^12^

In 1997, the UK National Confidential Enquiry into Patient Outcome and Death (NCEPOD) collected one week of data on all appendicectomies performed; 33% were by a senior house officer (SHO; equivalent of CT) and 42% by a JR.^13^ When this study was repeated in 2002, only 10.9% of paediatric appendicectomies were performed by SHOs and 18% by JRs.^14^ This suggests that appendicectomy, which used to be considered an SHO operation, is performed less and less frequently by this grade. Prioritising CTs and JRs to perform appendicectomies under the supervision of SRs is one way to ensure that as many trainees as possible can gain exposure and experience.

Case selection forms an important element of the ad hoc entrustment decision that takes place for each case. This describes the process undertaken for any potential TT case, in weighing up the learner's capabilities, the potential risk to the patient, the time of day, urgency of the case and availability of staff and theatre time.^15^ We found most cases took place during working hours, Monday to Friday, in children aged over five years, and two-thirds were normal or simple appendices. These differences were statistically significant. The high negative appendicectomy rate (15%) in our TT cases may be a reflection of case selection towards simple cases, as our overall negative appendicectomy rate is 9.6%.

Time was recognised as one of the essential components of intraoperative teaching, from semi-structured interviews with surgeon and trainee pairs.^16^ Feeling unpressured, and working with the same person were described as two contributing factors to successful intraoperative teaching. Median duration of laparoscopic appendicectomy was shown to be 72 minutes in one UK study and 67 minutes in an Italian study.^17,18^ A paper comparing TT with attending-supervised cases found a median duration of 65 minutes versus 56 minutes.^19^ Our median duration of surgery in TT cases was also 65 minutes, which suggests that near-peer teaching is still efficient for service provision.

Formal teaching or assessment to develop intraoperative teaching skills does not form part of surgical training in the UK. The Train the Trainers course is to be completed by the end of surgical training, so many JRs may not have received any formal training in teaching.^20^ Resident-as-Teacher (RaT) programmes in the US were present in only 26% of General Surgical training programmes, compared with greater than 80% in General Medicine and Paediatrics. This is despite 96% of US programme directors identifying teaching as an important aspect of a Resident's job.^21^ However, the American Board of Surgery has mandated a minimum of 25 cases of TT to complete training, recognising its importance.^22^ In the UK, whereas TT cases can be counted towards the total cases performed, there is no minimum requirement either in number of cases, or in feedback or workplace-based assessments to demonstrate ability to teach intraoperatively.

Only 0.7% of articles in leading medical education journals used patient outcomes as a measure of teaching performance,^23^ but this is arguably one of the most important factors to consider in adopting a teaching technique. Several papers have shown that allowing Registrars to operate independently does not lead to increased complications,^23–25^ but there are fewer studies examining the safety of TT. A prospective randomised study compared two groups of 60 laparoscopic appendicectomies performed by General Surgery junior residents (PGY1–3); group A was assisted by the attending, and group B by the senior resident. They found no significant difference in duration of hospital stay or complications.^19^ A retrospective review compared 597 General Surgery resident cases (operating with an attending) with 331 ‘teaching resident’ cases (two residents operating) and found no significant differences in rates of postoperative collection (8.2% vs 6.7%), conversion (2.9% vs 3%) or readmission within 30 days (5.9% vs 4.5%).^26^ Our complications were similar to this group, despite having a much higher rate of procedures performed without Consultant presence (78%). This paper's 35% rate of TT cases demonstrates the potential of TT as a teaching model—if we increased our TT cases from 20% to 35%, this would create an additional 37 teaching opportunities for trainees.

Our results are comparable with published data. In 2012, the UK Paediatric Surgery Trainee Research Network (PSTRN) published two months of national data on appendicectomies performed in children. A total of 242 appendicectomies were performed, with 66.1% started laparoscopically and a 5% conversion rate. The negative appendicectomy rate was 10.3%. They demonstrated a 15.3% rate of readmission within 30 days of discharge, and a 4.3% reoperation rate (7% and 1.1%, respectively, in our study).^27^ A US study of 3,393 Paediatric appendicectomies averaged 113 cases per centre per year, similar to our centre's 128 operative cases per year. This paper reported a low negative appendicectomy rate of 2.6%, although with a much higher use of computed tomography (14% in simple appendicitis and 12% in complicated cases). Their readmission rate was 4.3%.^28^

Of the 53 TT cases, only 8 were supervised by an unscrubbed Consultant. This could provide an opportunity for feedback on the teaching given by the Registrar, to maximise the learning experience. Another option could be asking the Consultant Anaesthetist to give feedback on teaching skills, which has been described in previous studies.^29^ One major drawback of this retrospective study is that detail on the quality of the learning is not possible to ascertain.

Further work could involve evaluating the benefits and drawbacks for the trainees, both the teacher and the learner, via questionnaires or structured interviews, such as that used by Papochristos *et al*.^30^ Video or direct observation of learning interactions in theatre with structured feedback from a Consultant Surgeon could aid assessment of the quality of teaching. In the UK surgical curriculum, the only existing work-based assessment specifically for teaching skills is called the Observation of Teaching (OoT), and is for ‘formal situation[s] where others are gathered specifically to learn from the speaker, and does not include…occasions of teaching in the presence of a patient’.^31^ Therefore OoT is not suitable for the purpose of intra-operative teaching; an alternative could be Objective Structured Teaching Evaluation (OSTE), which uses a simulated learner to assess teaching skills, and has been tested in Neurosurgical trainees teaching lumbar puncture and ventriculostomy.^32,33^ A study by Ann Arbor University showed that introducing a workshop specifically for Fellows (senior trainees) on intra-operative teaching led to an increase in their interest and confidence in teaching in this setting.^34^ In the UK, mandated minimum trainee as trainer cases in the operative logbook, as required in the US, would demonstrate our commitment to training future surgeons who can effectively teach intra-operative skills.

## Conclusion

Laparoscopic appendicectomy is an excellent model for trainees to act as trainer. Opportunities can be maximised by giving preference to CTs or JRs for appendicectomies supported by SRs, starting TT early in training, and using appropriate case selection for simpler cases, performed during daylight hours. Importantly, this is a safe teaching model for patients.
